# Fracture of the Os Brachii at the Insertion of the Deltoid Muscle in a Colored Preacher While in the Act of Gesticulating

**Published:** 1852-09

**Authors:** W. Parker

**Affiliations:** Professor of Surgery in the College of Physicians and Surgeons, New York


*Fracture of the Os Brachii at the insertion of the Deltoid Muscle in a Colored Preacher while in the act of Gesticulating.* By W. Parker, M. D., Professor of Surgery in the College of Physicians and Surgeons, New York.—I was called in consultation with Dr. Sargeant, in Jnly, 1844, in the case of Mr. R. Jackson, a colored minister of the Methodist denomination, who was suffering from a fracture of his right arm. He was of a stout build, very muscular and well developed, thirty-eight years of age, having no hereditary predispositions, and had never previously suffered a fracture of any of his limbs. There was no circumstance connected with his history giving evidence of unnatural fragility of his bones. The accident occurred while warmly engaged in an exhortation in a religious meeting, and upon making a violent gesticulation. On examination, the right humerus was found broken at the attachment of the deltoid. Union took place in the usual time.—*Ibid.*

